# Circular polarized 3D-printed cylindrical DRA using parasitic dielectric helix

**DOI:** 10.1038/s41598-023-39098-2

**Published:** 2023-07-22

**Authors:** Sebastian Diaz, Marcos Diaz, Eva Rajo-Iglesias, Francisco Pizarro

**Affiliations:** 1 Escuela de Ingeniería Eléctrica, Pontificia Universidad Católica de Valparaíso, 2362804 Valparaiso, Chile; 2 Space and Planetary Exploration Laboratory (SPEL), Electrical Engineering Department, Faculty of Physical and Mathematical Sciences, University of Chile, 8370448 Santiago, Chile; 3 Department of Signal Theory and Communication, University Carlos III of Madrid, 28911 Madrid, Spain

**Keywords:** Electrical and electronic engineering, Applied physics

## Abstract

This article presents a 3D-printed cylindrical dielectric resonator antenna operating at 5.8 GHz that achieves circular polarization by integrating a fully dielectric parasitic helix with a higher permittivity than the cylindrical resonator. The antenna polarization can be right-handed or left-handed depending on the turning sense of the helix. An extensive parametric study was done for the helix design to evaluate the effects of the dimensions and dielectric constant of the helix over the matching and axial ratio of the antenna. The manufacturing is made using low-loss dielectric filaments and a low-cost 3D printer. Simulation and measurement results show that both antennas are well-matched and operate with the corresponding circular polarization, with an axial ratio bandwidth compatible with UAV applications.

## Introduction

Circular polarization (CP) has been used in a wide variety of wireless communication applications, such as satellite communications and unmanned vehicles^1^, due to its many advantages. For example, when using CP the delay spread can be reduced, ensuring higher levels of received power^2^, and this polarization has a higher resistance to multipath interference^3,4^. Different methods can be found in the literature to obtain circular polarized antennas, which are mainly based on modifying the radiating structure or the antenna feed network^5,6^.

In the perspective of implementing CP antennas, circular polarized dielectric resonator antennas (DRA) can also be found, which can be interesting candidates for the mentioned applications, due to their versatility in terms of shapes, radiation patterns, and the possibilities of implementation. Nevertheless, DRAs can be limited when designing complex shapes if traditional dielectric manufacturing techniques are used, resulting in higher costs^7^. One technology that can overcome this issue is their implementation using additive manufacturing.

Additive manufacturing or 3D-printing is suitable for many applications in engineering^8^, including high-frequency topologies^9^ due to the availability of low-cost and low-loss dielectric filaments and high-precision 3D-printers. This made it possible to implement topologies with shapes that were either too expensive or impossible to implement without this technology^10^. Some examples of implementing DRAs using 3D printing can be found in the literature, such as high-gain structures^11^, multi-ring structures^12^, and the conference paper containing the preliminary work on the design presented here^13^.

The antenna presented in this article consists of the design, parametric study, modal analysis, implementation, and measurement of a cylindrical dielectric resonator antenna (DRA) operating at 5.8 GHz, which uses a parasitic dielectric helix with high permittivity to achieve circular polarization. The turning sense of the helix determines the sense of the polarization to be right-handed (RHCP) or left-handed circular polarization (LHCP).

## Antenna design and simulation results

**Figure 1:**
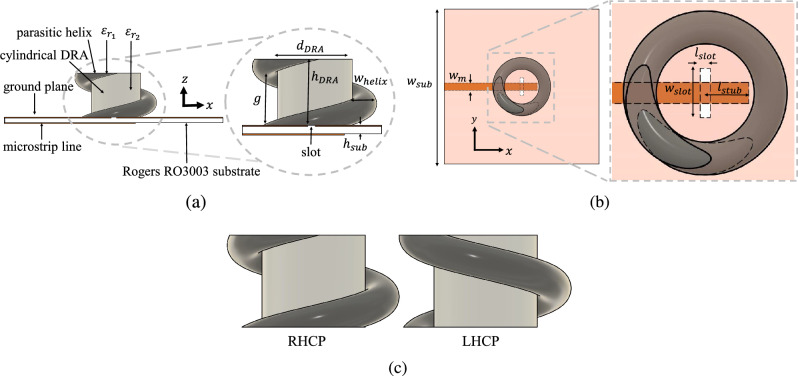
Proposed CDRA with helix and feeding structure. (**a**) Side-view (**b**) Top-view (**c**) Dielectric helix structure for LHCP and RHCP.

**Figure 2:**
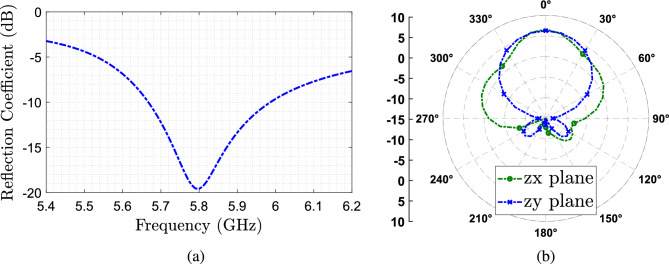
Simulated CDRA results. (**a**) $S_{11}$ as a function of the frequency (**b**) Radiation pattern at 5.8 GHz.

The proposed antenna is shown in Fig. 1. It consists of two dielectric structures: a cylindrical dielectric resonator antenna (CDRA) and a parasitic dielectric helix placed around the CDRA. First, the dimensions of the CDRA are designed for a resonance frequency $f_0$ at 5.8 GHz exciting the HEM$_{11\Delta }$ mode^14,15^, using a slot coupling feed^7^ defined by Eq. (1):

1 $$\begin{aligned} f_0 = \frac{c}{2\pi \varepsilon _r}\sqrt{2\left( \frac{\pi }{r_{DRA}} \right) ^2+\frac{\pi ^2}{2h_{DRA}}} \end{aligned}$$

where *c* is the speed of light, $r_{DRA}$ is the DRA radius ($d_{DRA}/2$) and $h_{DRA}$ the DRA height. The form factor selected for this DRA gives a dimension for the radio of $r_{DRA}=9$ mm and a height $h_{DRA}=16$ mm, using a material with relative permittivity $\varepsilon _{r2}=9$. In Fig. 2, the simulated $|S_{11}|$ of the designed CDRA, and the simulated radiation pattern for both planes at 5.8 GHz are shown. We can see that the CDRA has a maximum gain of around 6 dBi, while it is well matched at the design frequency.

Once we have correctly designed the CDRA, we proceed to the implementation of the dielectric helix that will give circular polarization to the original DRA. First, the helix curve equations are defined as a function of the parameters shown in Fig. 1. The equations are defined having a Cartesian reference plane (*x*, *y*, *z*)^16^ and presented for each axis in Eqs. (2), (3) and (4):

2 $$\begin{aligned} x= & {} \left( r_{dra} + \frac{w_{helix}}{2} \right) \cos {(t)} \end{aligned}$$

3 $$\begin{aligned} y= & {} \left( r_{dra} + \frac{w_{helix}}{2} \right) \sin {(t)} \end{aligned}$$

4 $$\begin{aligned} z= & {} \left( \frac{g}{2 \pi } \right) t \end{aligned}$$

where $w_{helix}/2$ is the radius of the helix, *g* defines the total separation or gap between each turn, while the total length of the helix is defined by *t*, which starts from $t_0 = 0$ to $t_f =2\pi h_{dra}/{g}$, which depends of the height of the DRA $h_{dra}$ and the separation between turns. For the design of the helix, a parametric study is done in order to get a circularly polarized antenna at 5.8 GHz. This study involves three possible design parameters for the helix: the helix width $w_{helix}$, the helix relative permittivity $\varepsilon _{r1}$, and the gap between turns *g*, which can be obtained by the helix pitch. Regarding the permittivity of the helix, two possible cases are defined for the study: a permittivity higher than the CDRA ($\varepsilon _{r_1}$ = 13) and using the same value as the CDRA ($\varepsilon _{r_1}$ = 9).

**Figure 3:**
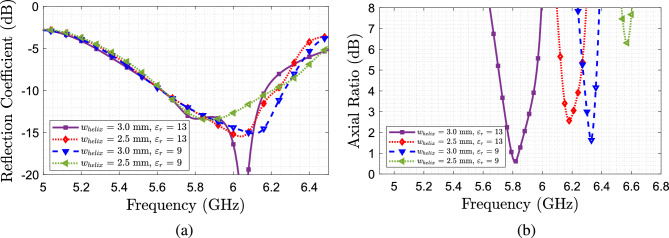
Parametric study considering different helix widths. (**a**) Axial ratio. (**b**) $S_{11}$.

**Figure 4:**
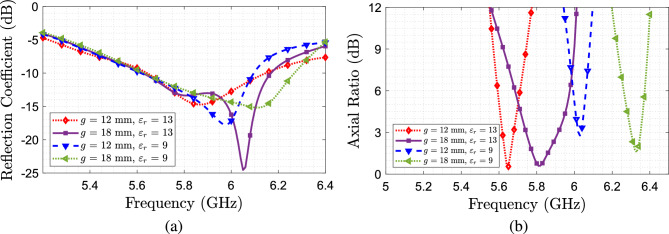
Parametric study considering different helix pitch. (**a**) *AR*, (**b**) $S_{11}$.

In Fig. 3 is shown the parametric study results using different values for the helix width and its impact over the reflection coefficient and the axial ratio. This study is done considering the two relative permittivities previously described, 13 and 9, and a helix gap of 18 mm. It can be seen that for all cases, the antenna remains matched on the frequency of interest, however, a shift of frequency and a degradation of the axial ratio occurs when reducing the relative permittivity and width of the helix. A second study of the variation of the gap between turns *g* is done by fixing the helix width at 3 mm. From the results shown in Fig. 4, we can see that when fixing the permittivity, a higher separation between turns leads to a larger AR bandwidth. From these studies, in conclusion, we need to use a larger permittivity value, for the helix, and a larger separation between turns, taking into account the width of the helix.

### Simulation results of the CDRA with parasitic helix

The proposed antenna following the model presented Fig. 1 has the following dimensions: $d_{DRA}=18$ mm, $h_{DRA}=16$ mm, $\varepsilon _{r2}=9$, $d_{helix}=18$ mm, $w_{helix}=6.0$ mm $g=18$ mm and $\varepsilon _{r1}=13$. For the feeding, a Rogers RO3003 ($\varepsilon _r=3$ and tan$\delta =0.0013$) substrate is used. The lateral dimensions and width of the substrate, as shown in Fig. 1 are $w_{sub}=80$ mm, and $h_{sub}=1.52$ mm, while the dimensions of the aperture on the slot feeding are $w_{slot}= 9.0$ mm, $l_{slot}= 1.8$ mm, $l_{stub}= 8$ mm and the width of the microstrip line is $w_m=3.82$ mm. Two implementations, the RHCP and LHCP structures, are simulated using ANSYS HFSS.

**Figure 5:**
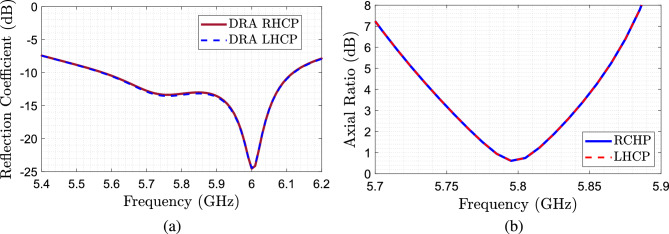
Simulated results of the antenna. (**a**) $S_{11}$ as a function of the frequency for the CDRA with helices. (**b**) Axial ratio for the RHCP and LHCP cases.

In Fig. 5 the simulated reflection coefficients $S_{11}$ of the CDRA with the parasitic helix and the corresponding axial ratios are shown. We can see that when the helix is present, we can clearly see two resonances, corresponding to the two excited orthogonal modes^17,18^, resonating at 5.7 GHz and 6.1 GHz. For the axial ratios, as expected, both antennas have the same axial ratio, independently of the polarization, reaching values below 3 dB at the operational frequency.

**Figure 6:**
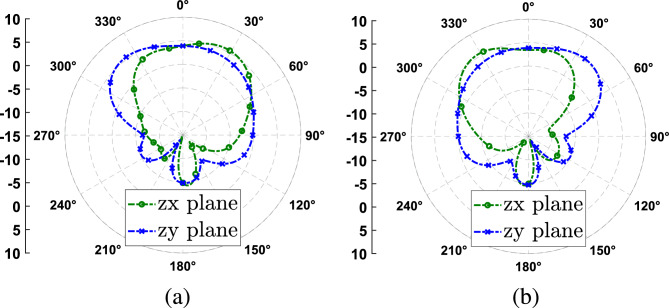
Simulated gain radiation pattern at 5.8 GHz in the two main planes and for both polarization cases. (**a**) RHCP antenna. (**b**) LHCP antenna.

**Figure 7:**
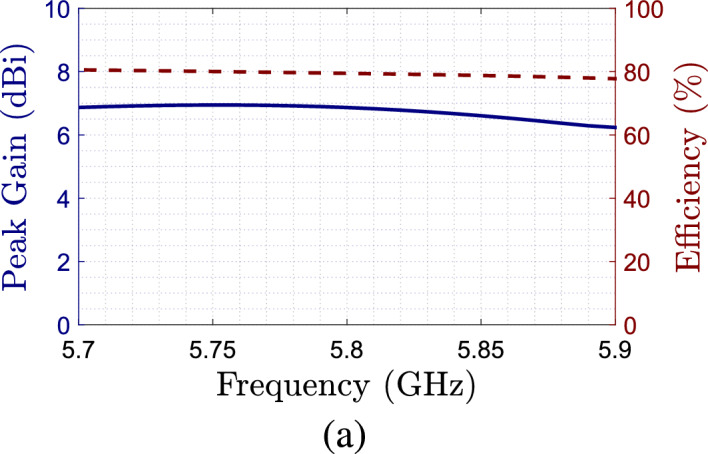
Simulated peak gain and efficiency as a function of the frequency.

In Fig. 6 the simulated gain radiation patterns at 5.8 GHz for both polarization are shown. We can see that the antenna radiation is slightly modified depending on the polarization, having a maximum gain of 5 dBi at the design frequency at $0^\circ$. Finally, Fig. 7 shows the simulated peak gain and antenna efficiency over the axial ratio bandwidth of the antenna. We can see that the antenna efficiency is around 80% over the assessed bandwidth, while the peak gain is between 6 and 7 dBi over the same band.

**Figure 8:**
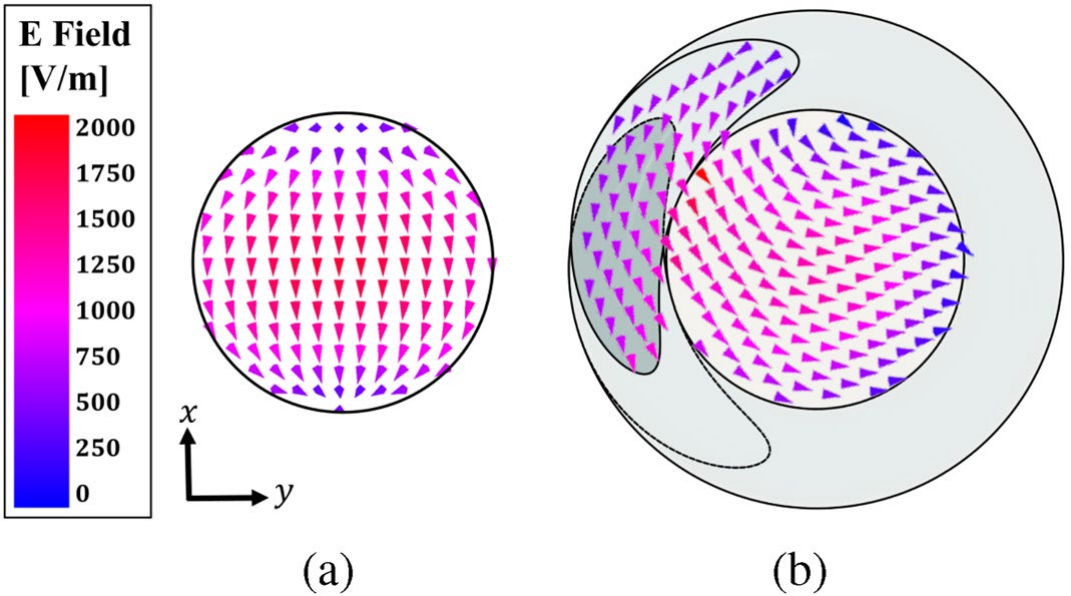
Simulated E-field vector distribution. (**a**) CDRA. (**b**) CDRA with RHCP parasitic dielectric helix.

**Figure 9:**
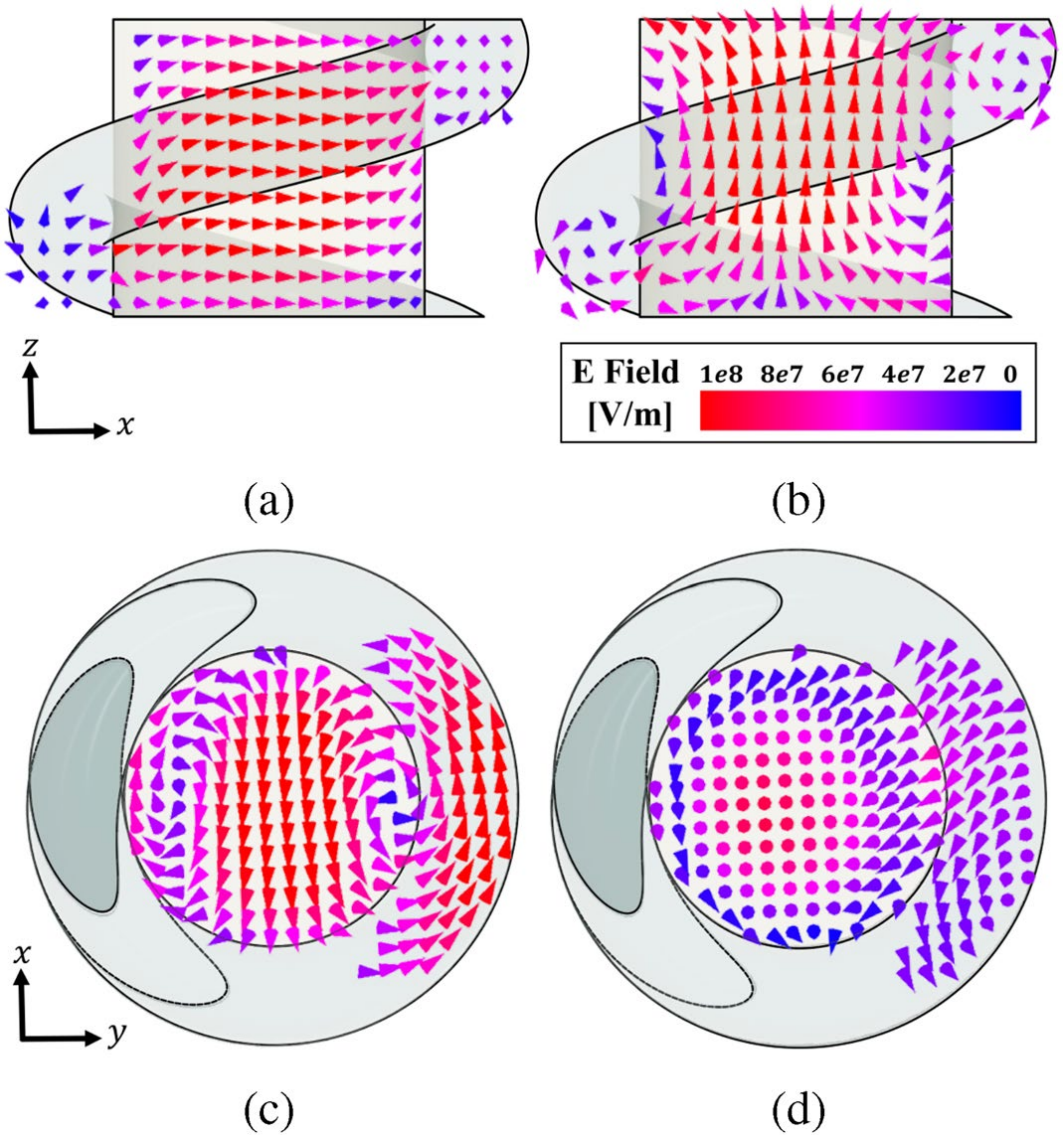
Simulated E-field vector distribution at the respective mode resonant frequencies. (**a**) Side view at 5.76 GHz, (**b**) Side view at 6.0 GHz, (**c**) Top View at 5.76 GHz, (**d**) Top View at 6.0 GHz.

It is important to identify the modes generated on the CDRA with the dielectric helix and check their orthogonality. The E-field vector distribution obtained with full-wave simulation is shown in Fig. 8. In the CDRA without the helix the expected HEM$_{11}$ mode is clearly seen, while when the helix is present, the electric field is following the RHCP behavior. To see and identify these two orthogonal degenerate modes, the DRA is simulated in the Eigenmode Ansys HFSS solution to obtain the fields in each resonant mode. The first mode is excited at 5.76 GHz as shown in Fig. 9 where we can identify it as a HEM$_{12\delta }$. The second mode is excited at 6.02 GHz, corresponding to the TM$_{01\delta }$ mode, being both orthogonal modes^19^. Based on this analysis, we expect to obtain a low axial ratio at the design frequency of $f_0=5.8$ GHz, similar to that achieved when designing circularly polarized (CP) patch antennas through the excitation of orthogonal modes.

## Antenna manufacturing using 3D-printing

Once the design is set, we proceed to manufacture the antenna using fused filament fabrication (FFF) 3D printing. The materials used for printing are the low-loss premix dielectric filaments from AVIENT^20^. For the helix, we use the ABS1500 filament, which has a $\varepsilon _{r_1}=15$, and for the CDRA we use the ABS1200 filament that has a nominal relative permittivity of $\varepsilon _{r_2}=12$. As for the 3D printer, we use a low-cost 3D printer from Ocular3D^21^ whose characteristics are summarized in Table 1.

**Table 1:** Custom 3D printer technical specifications.

Printer parameter	Value
Maximum printing volume	120$\times$120$\times$190 mm$^3$
Axis resolution	100 μm in all axis *(xyz)*
Nozzle diameter	0.4 mm
Filament diameter	1.75 mm
Hot-end T$^{\circ }$ range	120 to 260 $^{\circ }$C
Platform maximum T$^{\circ }$	100 $^{\circ }$C
Max. print speed	60 mm/s

**Figure 10:**
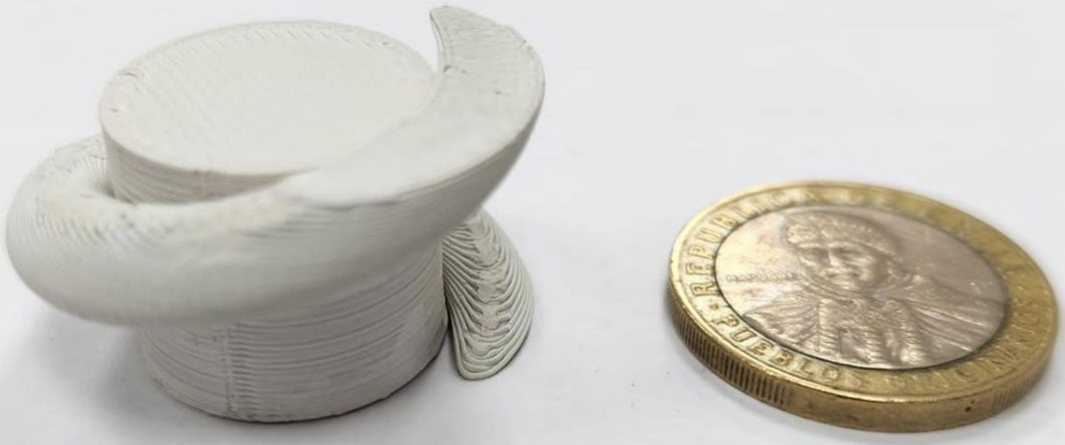
3D-printed RHCP DRA with helix.

The 3D-printing parameters used for the deposition of the filaments were set using a nozzle temperature of 260$^{\circ }$C, a bed temperature of 110$^{\circ }$C, a flux of 100%, and an infill percentage of 100%. Once the parameters are defined, we proceed to print samples and characterize their relative permittivity using the Nicolson-Ross-Weir method^22,23^ in the band of interest. It is known that there can be differences between the nominal value of the relative permittivity of the filament and the actual printed value depending on the printing parameters^24^, and therefore the characterization of samples is relevant for these implementations. The samples were characterized in the 4.9 GHz to 7.0 GHz band, using a WR159 standard waveguide. The resulting measured permittivities were $\varepsilon _{r_1}=13$ for the ABS1500 filament and $\varepsilon _{r_2}=9$ for the ABS1200 filament. Finally, the printed antenna is shown in Fig. 10.

## Measurement results

**Figure 11:**
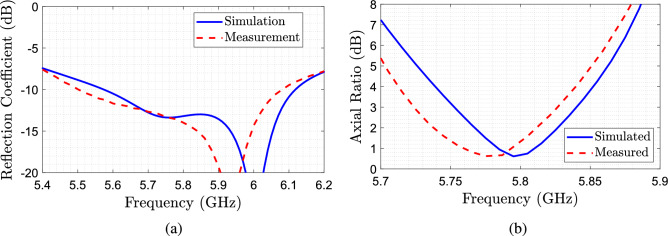
Measurement results of the RHCP designed antenna. (**a**) $S_{11}$ as a function of the frequency. (**b**) Axial ratio.

The reflection coefficient of the antenna was measured using a Vector Network Analyzer Anritsu MS46122B, while the gain radiation pattern and axial ratio were measured in an anechoic chamber. It should be noted that as the RHCP and LHCP implementations are identical in structure, we have only implemented the RHCP version for validation. Fig. 11 contains the simulated and measured reflection coefficient $|S_{11}|$ and the axial ratio as a function of the frequency of the DRA. We can see that the antenna is matched at the operational frequency, while the axial ratio reaches a value of around 1 dB at 5.8 GHz and an AR bandwidth of around 1.9%, obtaining a good agreement between measurements and simulations, but presenting a slight overall shift on the resonant frequencies. The slight frequency shift can be attributed mainly to the tolerances in the relative permittivity of the dielectric filament being used.

**Figure 12:**
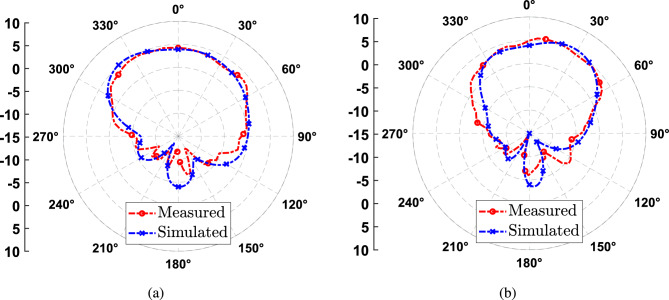
Comparison gain radiation pattern measured and simulated at 5.8 GHz. (**a**) ZY plane (**b**) ZX plane.

The simulated and measured gain radiation pattern in two cut planes of the DRA with the new dimensions are shown in Fig. 12. We can see a very good agreement with simulations, having a maximum gain of 5.3 dBi. Finally, a comparison with other CP single-fed DRA implementations is presented in Table 2. We can see that the helix implementation has a slightly larger ARBW, and the advantage is that it is 3D-printed using a low-cost 3D printer.

**Table 2:** Comparison between the CP-DRA implemented in this work with other single-fed CP-DRA implementations.

Structure	$f_c$ (GHz)	Feed type	ARBW (%)
Rectangular^25^	2.44	Probe	1.3
Hexagonal^26^	5.15	Slot	1.4
Rectangular^27^	5.4	Slot	1.8
Presented model	5.8	Slot	1.9

## Conclusion

The article presents a 3D-printed circular polarized cylindrical dielectric resonator antenna obtained by adding a parasitic dielectric helix to the structure. The design of this antenna is based on a simple CDRA, with a helix that gives the desired circular polarization depending on its turning sense, while the main characteristics of the antenna, such as maximum gain at the original resonance frequency and the matching at this same frequency remain close to the ones of the CDRA without the helix. In addition, the obtained axial ratio bandwidth is compatible with UAV applications. On the other hand, the possibility of fully implementing the structure using low-cost 3D printing makes it interesting for applications where cost, volume, and weight can be an issue, such as for unmanned vehicles. In addition, it is demonstrated how essential it is to characterize the printed filaments, as their final dielectric properties may vary depending on the printing parameters and printer characteristics. Finally, the versatility of this design makes it a good candidate topology to be tested using other types of DRA.

## Data Availability

The datasets used and/or analyzed during the current study are available from the corresponding author upon reasonable request.
